# Low-grade appendiceal mucinous neoplasms: a case series

**DOI:** 10.1093/jscr/rjaf214

**Published:** 2025-04-12

**Authors:** Mohammed N AlAli, Jawad S Alnajjar, Mohamed S Essa, Arwa F Alrasheed, Maha AlAmodi, Nouf A Alromaih, Sadiq M Amer, Abdulbaset Al-Shoaibi, Ameen M Alshehri

**Affiliations:** Department of Surgery, Prince Mohammed Bin Abdulaziz Hospital, Ministry of Health, Riyadh, Saudi Arabia; College of Medicine, King Faisal University, Alahsa, Saudi Arabia; Department of Surgery, Prince Mohammed Bin Abdulaziz Hospital, Ministry of Health, Riyadh, Saudi Arabia; General Surgery Department, Faculty of Medicine, Benha University, Benha Egypt; Department of Surgery, Prince Mohammed Bin Abdulaziz Hospital, Ministry of Health, Riyadh, Saudi Arabia; Department of Surgery, College of Medicine, King Khalid University, Abha, Saudi Arabia; Department of Surgery, Prince Mohammed Bin Abdulaziz Hospital, Ministry of Health, Riyadh, Saudi Arabia; Department of Pathology, Prince Mohammed Bin Abdulaziz Hospital, Ministry of Health, Riyadh, Saudi Arabia; Department of Radiology, Prince Mohammed Bin Abdulaziz Hospital, Ministry of Health, Riyadh, Saudi Arabia; Department of Surgery, Prince Mohammed Bin Abdulaziz Hospital, Ministry of Health, Riyadh, Saudi Arabia

**Keywords:** appendiceal tumors, mucinous neoplasms, cytologic atypia, appendectomy, cytoreductive surgery, pseudomyxoma peritonei

## Abstract

Appendiceal mucinous neoplasms, including low-grade appendiceal mucinous neoplasms (LAMNs), are infrequent but clinically relevant due to their potential progression to pseudomyxoma peritonei. Timely diagnosis and surgical intervention are critical to preventing complications. We present a case series of three middle-aged men with LAMNs, each of whom exhibited right lower quadrant abdominal pain as their primary symptom. Diagnostic imaging in each case revealed features consistent with appendiceal mucoceles. Pathological findings confirmed LAMN, with varying extents of disease. One patient underwent an open appendectomy for LAMN confined to the appendix, while two required right hemicolectomy due to perforation and extra-appendiceal mucin involvement. LAMNs can be managed effectively through early detection and surgical resection. Complete resection with negative margins results in favorable outcomes and low recurrence rates. More aggressive treatments, including cytoreductive surgery with or without hyperthermic intraperitoneal chemotherapy, are necessary in cases with peritoneal involvement.

## Introduction

Appendiceal mucinous neoplasms are a diverse group of tumors with an increasing incidence [1]. These neoplasms are found in 0.9%–1.7% of appendectomy specimens [2, 3]. They are categorized into high-grade appendiceal mucinous neoplasms (HAMNs) and low-grade appendiceal mucinous neoplasms (LAMNs) variants, with their distinction reliant on the histopathological degree of epithelial dysplasia [4].

HAMNs are classified as invasive adenocarcinomas owing to their elevated recurrence potential, whereas LAMNs exhibit a more uniform histological architecture, characterized by papillary or flat mucinous growth patterns with minimal cytologic atypia, resembling the low-grade dysplasia found in other gastrointestinal sites [4, 5]. Although LAMNs are considered morphologically benign, they still carry the risk of progressing aggressively to pseudomyxoma peritonei (PMP) [6].

This highlights critical considerations regarding the optimal management approach for LAMNs when identified in surgical specimens, which are frequently excised due to diverse clinical presentations, including acute appendicitis, presumed ovarian mass, or incidental detection of a mucocele on imaging [6]. This case series evaluates three LAMN cases, discussing their demographics, presentation, diagnosis, and management.

## Case presentation

### Case 1

**Figure 1:**
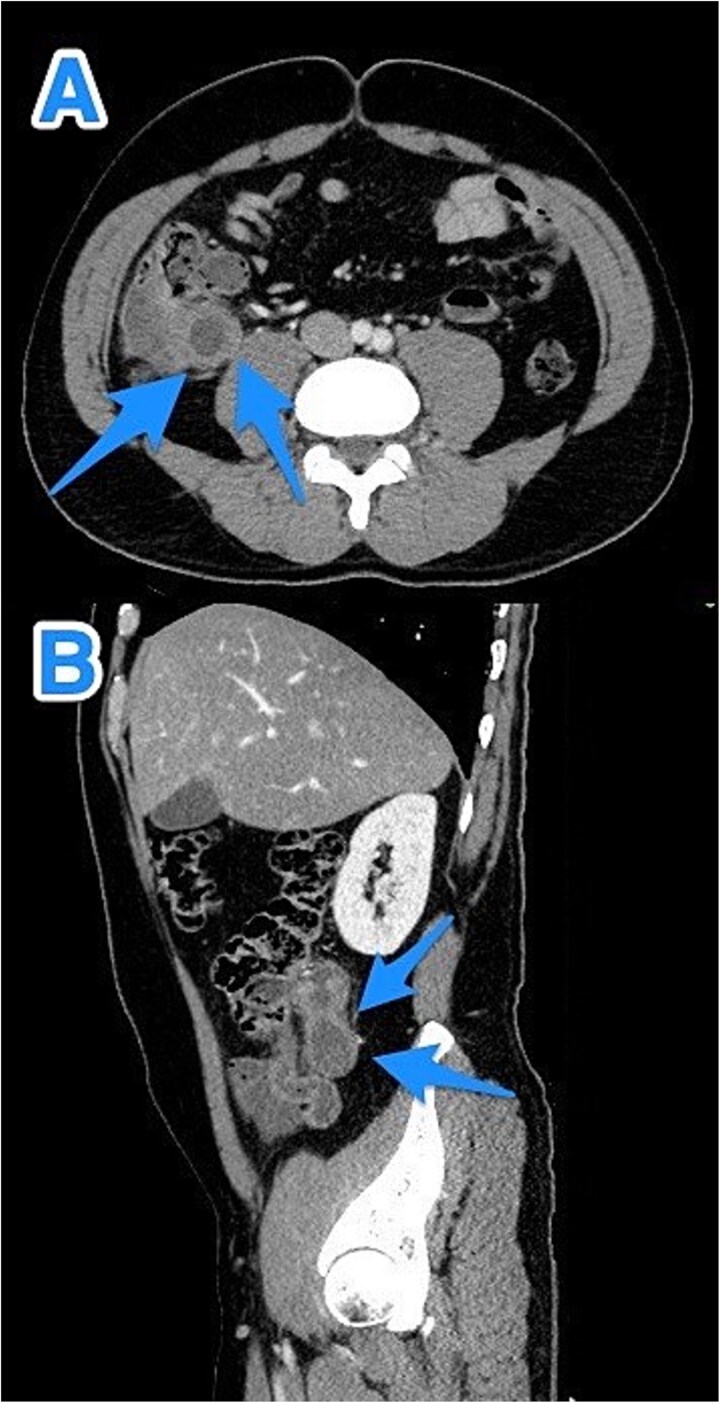
CT abdomen and pelvis with contrast—Axial (A) and sagittal (B) views highlighting a dilated, fluid-filled appendix with a maximum diameter of 2.2 cm and small peripheral calcifications (arrows).

**Figure 2:**
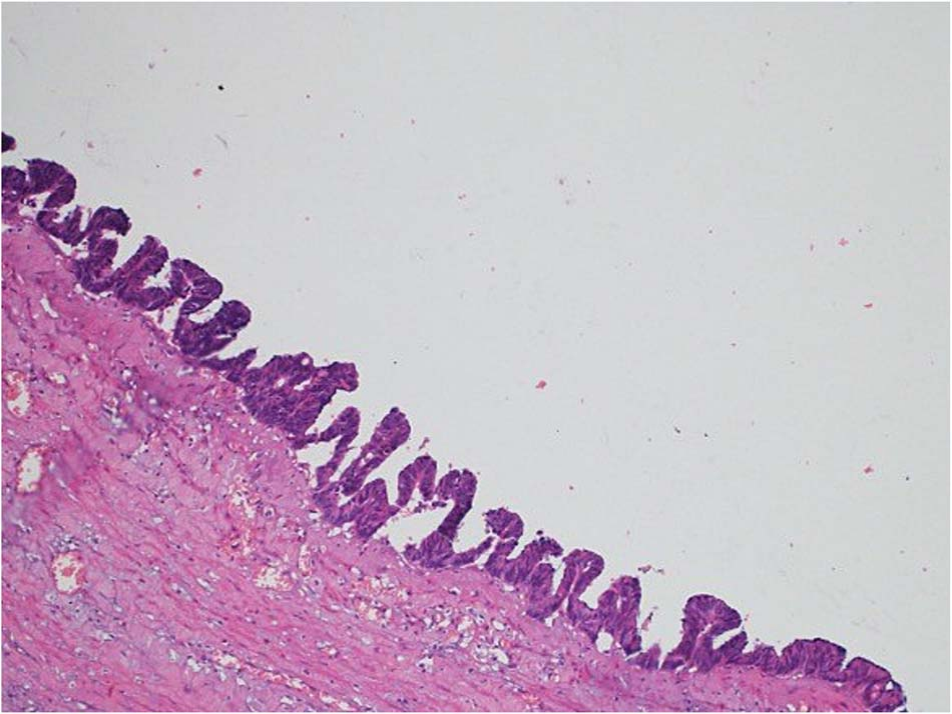
Light microscopy images of the appendix show low-grade dysplastic epithelium on the surface of the appendix (hematoxylin and eosin stain; 10×).

A 47-year-old male patient, with a documented history of diabetes mellitus but no previous surgical interventions, presented with right lower quadrant (RLQ) abdominal pain, which was associated with recurrent vomiting episodes (four times) within the preceding 24 h. The pain did not radiate and was not aggravated by any specific factors. The patient denied fever, urinary symptoms, and changes in bowel habits. On physical examination, he appeared well, with mild pain but no distress. Abdominal examination revealed minimal tenderness and rebound tenderness in the RLQ, but the abdomen was otherwise soft and lax, with no evidence of guarding, rigidity, or fullness. The patient’s laboratory profile was within normal ranges. Abdominal computed tomography (CT) with contrast revealed a dilated, fluid-filled appendix with a maximum diameter of 2.2 cm and tiny peripheral calcifications. There was no surrounding fat stranding or free fluid. The rest of the bowel loops were grossly unremarkable (Fig. 1). A diagnosis of appendicular mucocele was made, and the patient underwent an open appendectomy. The patient’s postoperative course remained clinically uneventful. Gross findings of the appendectomy specimen measured 6 × 2.5 × 2 cm, with a smooth outer surface and no identified perforation. Pathology examination revealed LAMN, confined to the appendix. The sections showed flat mucinous epithelium originating from the lumen, and there was no evidence of extra-appendiceal mucinous extension. The margin was negative for mucinous neoplasm (Fig. 2). A referral to the Colorectal Surgery Clinic was arranged to ensure a thorough evaluation and appropriate follow-up.

### Case 2

**Figure 3:**
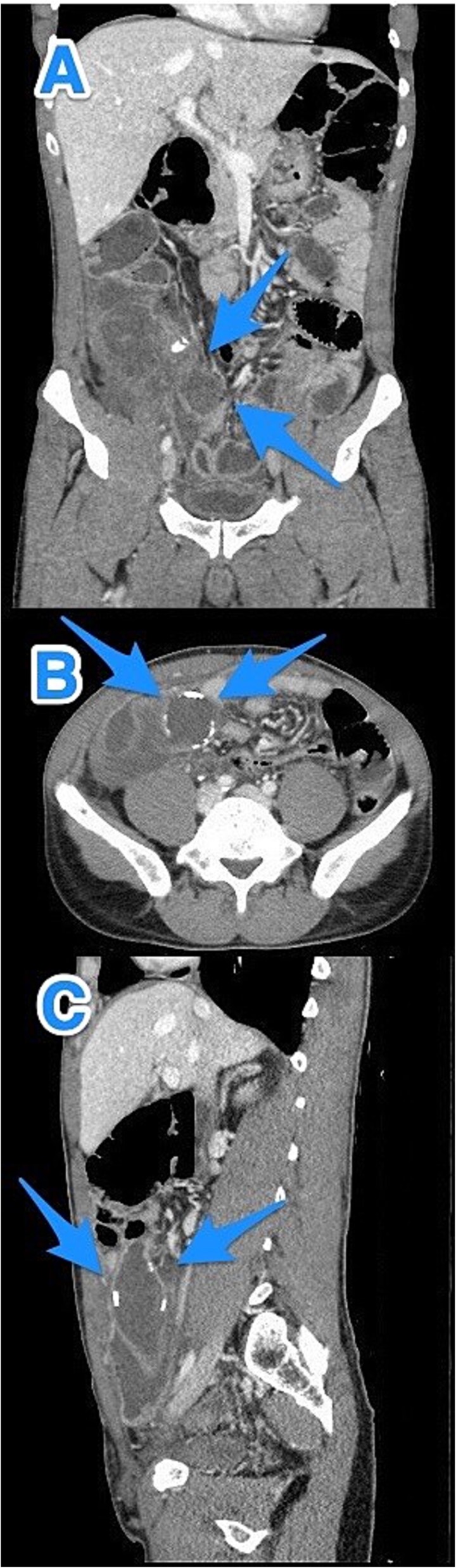
CT abdomen and pelvis with contrast: (A) coronal, (B) axial, and (C) sagittal views demonstrate a dilated, blind-ended structure in the right lower quadrant with incomplete circumferential calcifications and thick fluid content, consistent with an appendiceal mucocele (arrows).

A 46-year-old male, with no previous medical or surgical history, presented to the emergency department with a 4-day history of right lower abdominal pain, which initially originated in the epigastric region before migrating to the right iliac fossa. It was constant, progressive, and not relieved by simple analgesics. The patient reported associated nausea and anorexia but denied any history of fever or vomiting. On physical examination, the patient was vitally stable, alert, conscious, and in moderate pain. There was rebound tenderness in the RLQ, and the remainder of abdomen was soft, with no other positive findings. Laboratory investigations were within normal ranges. Contrast-enhanced CT imaging of the abdomen and pelvis detected a dilated, blind-ended tubular structure in the right lower quadrant, with thick fluid content and incomplete calcifications along its circumference. A focal area of wall discontinuity with thick fluid collection in the right paracolic gutter and pelvis was observed (Fig. 3). Additionally, small ascites with thickened and enhancing peritoneal reflections were noted. The diagnosis of a ruptured appendiceal mucocele was made. A right hemicolectomy was performed, and the patient's postoperative course remained stable and uneventful.

**Figure 4:**
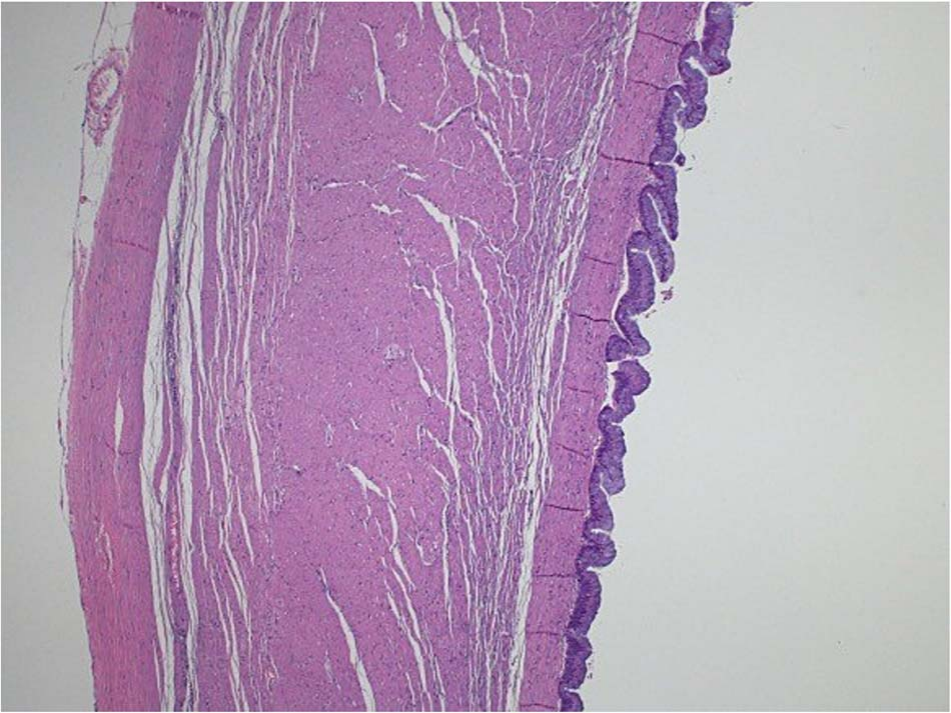
Light microscopy images of the appendix show low-grade dysplastic epithelium on the surface of the appendix (hematoxylin and eosin stain; 10×).

Pathological examination confirmed the diagnosis of LAMN (Fig. 4). Key findings included tumor disruption of the appendiceal wall, pushing invasion into the muscularis propria, and associated perforation. There was mucin dissection extending into the extra-appendiceal region, accompanied by a serosal reaction characterized by granulation tissue and acute inflammation. Both resection margins were tumor-free, and all 15 lymph nodes examined were negative for tumor infiltration. A focus of lymphovascular invasion was identified. Biopsies of the peritoneum and omentum demonstrated mucin dissection, extensive acute serositis, and inflammation. A referral to a higher center was initiated for comprehensive assessment and ongoing medical management.

### Case 3

**Figure 5:**
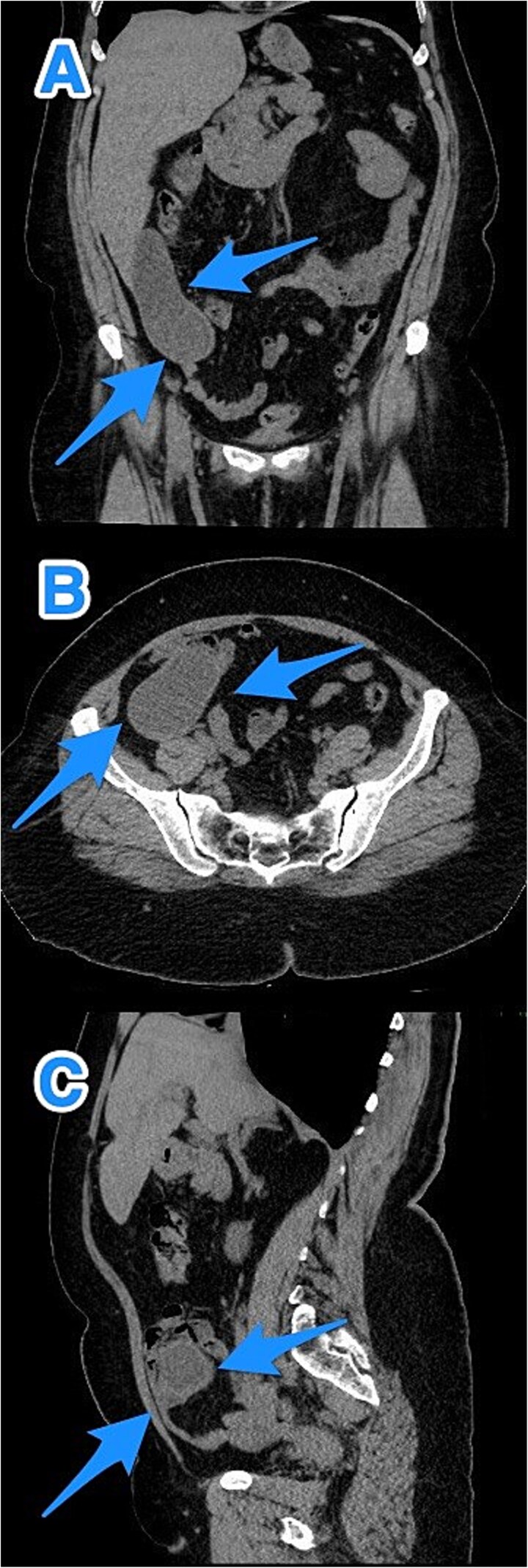
CT abdomen and pelvis with contrast: (A) coronal, (B) axial, and (C) sagittal views reveal an appendix measuring 22 cm in the right iliac fossa, with edematous wall thickening, minimal surrounding fat stranding, and an adjacent fluid collection measuring 3.3 × 1.8 × 8.5 cm, consistent with a perforated appendix (arrows).

**Figure 6:**
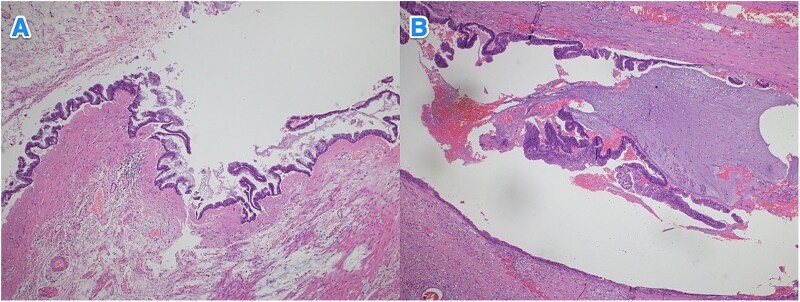
Light microscopy images of the appendix (A, B) show low-grade dysplastic epithelium on the surface of the appendix (hematoxylin and eosin stain; 4×).

A 46-year-old male with a history of leukemia treated with chemotherapy and primary infertility presented with right lower quadrant abdominal pain of less than one day's duration. He reported associated nausea but no vomiting, changes in bowel habits, or fever. On systemic evaluation, the patient demonstrated stable vital signs, full consciousness, and alertness, with no evidence of severe pain. Abdominal examination revealed rebound tenderness in the RLQ, but the abdomen was otherwise soft, with no tenderness upon palpation of other regions. Laboratory investigations were within normal limits. Abdominal CT with contrast revealed evidence of appendiceal perforation. The liver showed moderate to severe steatosis, but no other remarkable abnormalities were detected (Fig. 5). Due to the perforation near the base of the appendix, a right hemicolectomy was performed. Histopathological examination of the appendix confirmed the diagnosis of LAMN (Fig. 6). The tumor extended to the visceral peritoneum through the perforation site, but there was no evidence of lymphatic, vascular, or perineural invasion. Four reactive lymph nodes were negative for tumor infiltration, and the pathologic TNM staging was pT4, pN0. The specimen also contained acellular mucin evacuated during the surgery. Postoperative recovery was uneventful, and the patient was later transferred to a higher center for comprehensive assessment and continued care.

## Discussion

LAMNs that remain confined to the appendiceal wall and serosal surface, without evidence of peritoneal dissemination at the time of diagnosis, are not associated with a significant risk of disease progression [6]. In contrast, cases with peritoneal disease involving neoplastic low-grade epithelium are at a significantly higher risk [6]. Several risk factors for LAMN have been proposed, including female gender, Crohn’s disease, appendiceal perforation, the presence of anemia upon admission, and prolonged symptom duration [7]. Notably, all three cases reported in this study involved male patients and there was not coleration with aforementioned risk factors. Diagnosis can be achieved through ultrasonography, CT scan, or magnetic resonance imaging (MRI) [8]. Tumor markers, such as carcinoembryonic antigen (CEA) (elevated in 56.1% of cases) and Ca 19-9 (elevated in 67.1% of cases), are frequently elevated [9], However, they were not available at out hospital.

In the management of LAMN, if the appendix is not perforated, appendectomy is the recommended treatment. Even in the case of perforation with peritoneal mucin, appendectomy remains advised. However, if perforation with peritoneal cellular mucin occurs, more aggressive treatment, involving cytoreductive surgery with or without hyperthermic intraperitoneal chemotherapy (HIPEC), is necessary [10]. An active surveillance strategy using MRI has also been proposed as a valid option after an incidental LAMN diagnosis [11]. Complete excision of LAMNs or HAMNs with negative surgical margins yields very low recurrence rates, consistent with their relatively non-aggressive behavior. When LAMNs are resected with a positive margin, studies suggest that right colectomy may not be needed, as recurrence remains rare [12]. Over a 5-year period, recurrence-free survival has been documented at 95.1%, despite the fact that ˃50% of resected specimens demonstrated rupture or extra-appendiceal mucin. Given the low recurrence incidence, the value of routine postoperative imaging remains minimal. Nevertheless, MRI of the abdomen every 6 months for the first 2 years postoperatively is advised, as recurrences, when present, typically emerge early [12]. Lohani *et al.* identified that recurrence was significantly associated with LAMNs confined to the right lower quadrant and with tumor sizes ˂2 cm [13].

LAMNs exhibiting peritoneal deposits of acellular mucin demonstrate an intermediate potential for disease advancement, aligning with the updated staging criteria for these neoplasms [7]. The overall survival rates for LAMNs with extra-appendiceal spread are 100%, 86%, 60%, and 45% at 3, 5, 7, and 10 years, respectively [1]. PMP progression was noted in 20% of patients post-appendectomy, evidenced by diffuse mucinous peritoneal implants and extensive gelatinous intra-abdominal collections [14]. PMP progression leads to abdominal distension and bowel obstruction [15]. Appendiceal tumor variables, including perforation, marginal positivity, and extra-appendiceal deposition of acellular mucin or mucinous epithelium, were not identified as significant predictors of disease progression [14]. The combination of HIPEC after cytoreduction for appendiceal PMP may improve survival in low-grade cases, which have very favorable outcomes, while high-grade pathological disease may be linked to poor outcomes and rapid progression [16, 17].

## Conclusions

LAMNs are typically indolent but have the potential to progress to PMP if left untreated. Early and accurate diagnosis through imaging and histopathology is essential for optimal management. While appendectomy remains the standard treatment, cases involving perforation or cellular mucin may require more aggressive approaches, including cytoreductive surgery with or without HIPEC. Complete surgical resection offers the best long-term outcomes, but vigilant postoperative surveillance is crucial, particularly within the first 2 years, to effectively detect and manage potential recurrences.
